# Therapy Follows Diagnosis: Old and New Approaches for the Treatment of Acute Porphyrias, What We Know and What We Should Know

**DOI:** 10.3390/diagnostics12071618

**Published:** 2022-07-03

**Authors:** Petro E. Petrides

**Affiliations:** 1 Hematology Oncology Center & EPNET-Center for Acute Porphyrias Munich, Zweibrückenstr. 2, 80331 Munich, Germany; petrides@onkologiemuenchen.de or petro.petrides@lmu.de; Tel.: +49-89-229009; Fax: +49-89-229448; 2 Munich School of Medicine, Ludwig Maximilians University (LMU), 80539 Munich, Germany

**Keywords:** heme, acute intermittent porphyria, variegate porphyria, glucose effect, panhematin, heme arginate, givosiran, 5-ALA

## Abstract

Heme, iron protoporphyrin IX, is one of life’s most central molecules. Hence, availability of the enzymatic machinery necessary for its synthesis is crucial for every cell. Consequently, inborn errors of porphyrin metabolism that compromise normal synthesis, namely the family of porphyrias, undermine normal cellular metabolism given that heme has functions in catalytic centers, signal transduction and functional regulation and its synthesis is fully integrated into the center of intermediary metabolism. Very often, diagnosis of porphyrias is difficult and therefore delayed. Therapy can be as complicated. Over the last 50 years, several strategies have been developed: because of its integration with other parts of intermediary metabolism, the infusion of glucose (glucose effect) was one of the first attempts to counterbalance the dysregulation of porphyrin synthesis in porphyrias. Since heme synthesis is impaired, infusional replacement of heme was the next important therapeutic step. Recently, siRNA technology has been introduced in order to downregulate 5-ALA-synthase 1, which contributes to the patho-physiology of these diseases. Moreover, other novel therapies using enzyme protein replacement, mRNA techniques or proteostasis regulators are being developed.

## 1. General Aspects (Introduction)

### 1.1. Heme—Its Role in Human Metabolism

Iron in the form of the protoporphyrin IX complex plays a dominant role in human metabolism. It occurs in two redox states, the **hemin** Fe (III) complex or ferric protoporphyrin IX and the **heme** Fe (II) complex or ferrous protoporphyrin IX. Very often, however, the word heme is used to describe both ferrous and ferric forms of iron protoporphyrin IX. The heme iron complex is practically insoluble in aqueous solutions such as blood plasma and its propensity to generate reactive oxygen species (ROS) makes it toxic to many biomolecules.

**Figure 1:**
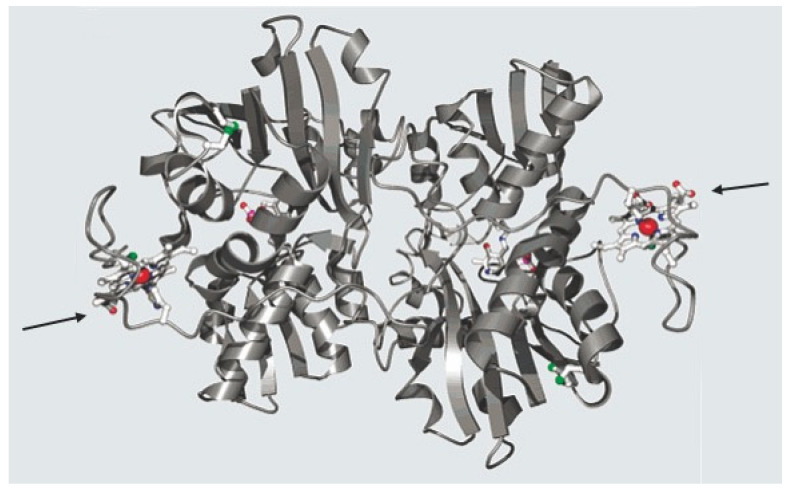
Human cystathionine-ß-Synthase (a Homotetramer): the protein backbone of a dimer is represented as ribbons, while heme (in a pocket, see arrows), pyridoxal phosphate and the so-called CXXC oxidoreductase motif are drawn as ball and stick models. The iron in the porphyrin moiety is shown as a red dot. (adapted from [4]) Copyright 2002 with permission from American Chemical Society).

In proteins, the bound heme iron complex plays important roles such as oxygen transport in hemoglobin or storage in myo- or neuroglobin, mediation of mitochondrial electron transport (for instance, cytochrome c) or degradation of drugs (cytochrom P450 = CYP enzymes). Moreover, certain enzymes such as catalases, peroxidases (such as myeloperoxidase) or NO-synthases also use heme as cofactors. In recent years, new roles of the heme iron complex have emerged: such as sensing of gases (carbon monoxide, nitric oxide, oxygen) through a permanently bound iron heme as a prosthetic group in a regulatory domain [1,2,3]. Sensing of the gas leads to a change of the activity of the catalytic domain. In human beings, for instance, cystathionine ß-synthase, which is involved in homocysteine metabolism through the condensation of serine and homocysteine, can be regulated by gases that bind to heme and lead to changes in enzyme activity (Figure 1). Another example is soluble guanylate cyclase, which is the human receptor of nitric oxide (NO) in numerous kinds of cells and produces the second messenger 3′,5′-cyclic guanosine monophosphate (cGMP) upon NO binding to its heme. NO is produced by NO-synthases, (see above) which convert arginine to citrulline with the concomitant release of NO. Heme oxygenases (I and II) catalyze the degradation of heme to biliverdin, CO and ferrous iron.

In addition, the binding of heme to transcription regulatory factors switches on and off the production of various enzymes and proteins that are critical for cell survival such as BACH1, tumor suppressor gene p53 or rev-erb-alpha and -beta as well as potassium channel proteins (Table 1).

### 1.2. Heme Biosynthesis and Degradation

Because of the paramount importance of heme for the wellbeing of a cell, probably all human cells can synthesize this molecule. While 80% (about 300 mg) of the body heme is made in the bone marrow for hemoglobin, about 15% (about 50 mg) is produced in the hepatocytes [5].

**Figure 2:**
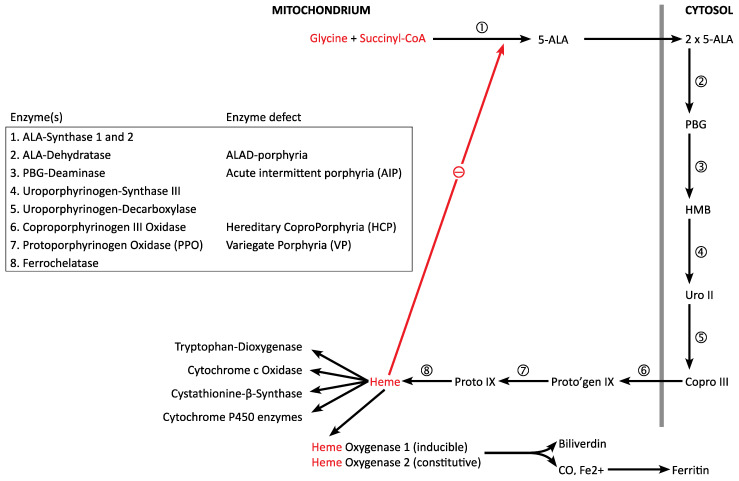
Biosynthesis of heme: the first four enzymes occur in tissue specific isoenzyme forms. d-ALA = d-aminolevulinic acid, PBG = porphobilinogen, HMB = hydroxymethylbilane, Uro III = Uroporphorinogen III, Copro III = Coproporphorinogen III, Proto = Protoporphyrin (ogen) IX. The enzyme defects in the four acute porphyrias are shown.

Heme is synthesized in an eight-step enzymatic process; of these the first and the last three steps occur in the mitochondrium, the other four in the cytosol (Figure 2). Starting with **succinyl-CoA**, an intermediate product of the tricarboxylic acid (TCA) cycle (located in the mitochondrium) and **glycine**, the most simple amino acid, the first metabolite 5-aminolevulinic acid (5-ALA) is formed. For this reaction to take place glycine has to be taken up from the cytosol through a transport system. The enzyme responsible for the catalysis is **5-ALA-Synthase** (5-ALA-S), which is the rate limiting step of the heme synthesis. In the liver and other cells, the house keeping isoenzyme 5-ALA-synthase 1 (5-ALAS1) is inhibited by the end product heme. In the bone marrow, the erythroid specific isoenzyme 2 (5-ALAS2, encoded by a different gene) underlies a different type of regulation. Since succinyl-CoA is continuously removed from the TCA cycle in a cataplerotic reaction, anaplerotic sequences are necessary to replenish the pools of metabolic intermediates (anaplerotic sequence) [6].

Next, 5-ALA travels from the mitochondrium into the cytosol, where two molecules 5-ALA condense to porphobilinogen (PBG) catalysed by **5-ALA dehydratase**. In human beings, two different enzymes (with different exons 1 in non-erythroid and erythroid cells) are produced from one single gene. In addition, in position 59, a polymorphism (resulting in a LYS-ASN variation) has been observed. The third enzyme in heme biosynthesis is **porphobilinogen deaminase**, which catalyzes the condensation of four molecules PBG to the linear tetrapyrrole hydroxymethylbilane (HMB). Like 5-ALA-dehydratase, two tissue specific forms of PBG-deaminase are produced from a single gene by alternate splicing.

The next step is the intramolecular rearrangement and ring closure of HMB with the formation of uroporphyrinogen III. Again, the enzyme in charge, **uroporphyrinogen synthase III (UROS)**, is present in two forms, due to alternate splicing. Upon decarboxylation with the formation of methyl groups, the more hydrophobic coproporphyrinogen III is formed. In this case, the responsible enzyme **uroporphyrinogen decarboxylase** (UROD) is present in only one isoform. After translocation into the mitochondrium, coproporphyrinogen III is converted through decarboxylation and oxidation into protoporphyrinogen IX by coproporphyrinogen oxidase (CPO), present only in one form in human tissues. With a second oxidation, protoporphyrin IX is produced by **protoporphyrinogen oxidase (PPO)**. In the last step, ferrous iron (Fe^2+^) is incorporated by **ferrochelatase** with the formation of the end product heme.

Degradation of heme occurs through heme oxygenases I (inducible) and II (constitutively active), which convert heme to biliverdin, CO and ferrous iron, which can be stored as ferritin [7].

**Figure 3:**
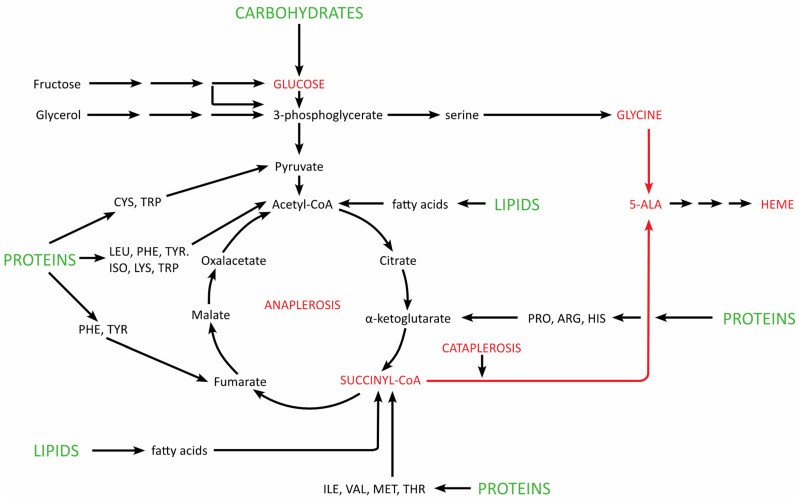
Integration of heme biosynthesis into carbohydrate, lipid and protein metabolism. Heme synthesis uses succinyl-CoA (cataplerosis), which is replenished by the TCA cycle (anaplerosis).

The heme biosynthetic pathway is fully integrated into the network of intermediary metabolism: succinyl-CoA is formed in the TCA cycle from alpha-ketoglutarate. Hence stimulation of heme biosynthesis requires an acceleration of the TCA cycle, which derives acetyl-CoA from the degradation of glucose, fatty acids and ketogenic as well as glucogenic amino acids (Figure 3). Glycine is produced from serine, which derives from 3-phosphogylcerate, an intermediary product of the glycolytic chain.

### 1.3. Regulation of Heme Biosynthesis and Degradation (“Hemeostasis”)

Heme production is determined by the needs of the cell. Here, I focus upon the regulation in the liver cell, which requires heme primarily for the synthesis of the family of CYP enzymes [8]. These enzymes are in charge of the detoxification of drugs, food ingredients (also from herbs) and additives as well as environmental contaminations. Since 5-ALAS-1 activity is the lowest among all heme biosynthetic enzymes, it is the rate- limiting step. As the end product, heme in the mitochondrium inhibits 5-ALA-synthase 1 which is in close proximity (feedback inhibition). Moreover, it can inhibit nuclear transcription and translocation of the 5-ALAS1-mRNA into the cytosol, translation on the ribosome and translocation of the 5-ALAS1-precursor into mitochondrium [9]. The system has a high redundancy, most likely due to the fact that heme overproduction would be toxic (see above).

On the other hand, heme oxygenase I (HO 1) can be induced by its substrate heme. Moreover, HO expression responds to many other diverse agents, such as oxidants, nitric oxide, heavy metals or plant derived polyphenolic compounds, to name a few [7,10].

### 1.4. The Acute Porphyrias: Disorders of Dysregulation of Heme Biosynthesis

Inborn errors of heme biosynthesis cause the acute porphyrias: heterozygous mutations in PBG-deaminase gene (enzyme 2 in Figure 2), coproporphyrinoxidase gene (enzyme 6 in Figure 2) and proto-porphyrinoxidase gene (enzyme 7 in Figure 2) cause acute intermittent porphyria (AIP), hereditary coproporphyria (HCP) and variegate porphyria (VP). These are autosomally transmitted and much more common than 5-ALA-Dehydratase porphyria, which requires mutations in both alleles (recessive inheritance) to become clinically manifest. Penetrance is low (10–20%) in the three dominant porphyrias, but disease modifier genes have not been identified yet [11].

It is generally assumed that the reduction of the enzymatic activity to 50% in steps 3, 6 or 7 is compensated through the stimulation of 5-ALAS1 activity, since a decrease of the concentration of the end product heme will lead to a feedback stimulation in liver and other cells (such as brain or muscle). However, no systematic metabolomic studies have been carried out to support this assumption. On the contrary, studies on the metabolism of homocysteine (which requires the activity of the heme-dependent enzyme cystathionine-ß-synthase) in acute porphyria patients have revealed a dysregulation, even in asymptomatic gene carriers [12,13].

Moreover, targeted metabolomic analyses have shown disturbances of the heme-dependent tryptophan metabolism (tryptophan dioxygenase) [14].

These observations may only be the tip of the iceberg, indicating that heme deficiency in patients may cause unexpected metabolic derangements.

### 1.5. The Acute Attack: Pathophysiology and Diagnosis

The acute porphyrias are—as the name implies—characterised by acute attacks: these occur when the demand for heme exceeds the capacity of the impaired heme biosynthetic pathway. Heme demand can be increased by all factors that induce CYP enzymes in the liver: this not only includes drugs but also steroid hormones, foodstuff or medicinal herbs or changes in metabolism such as starving (see below). In theory, also inducers of heme oxygenase I should also increase the risk of an attack in gene carriers since the heme pool has to be replenished through acceleration of heme biosynthesis.

**Figure 4:**
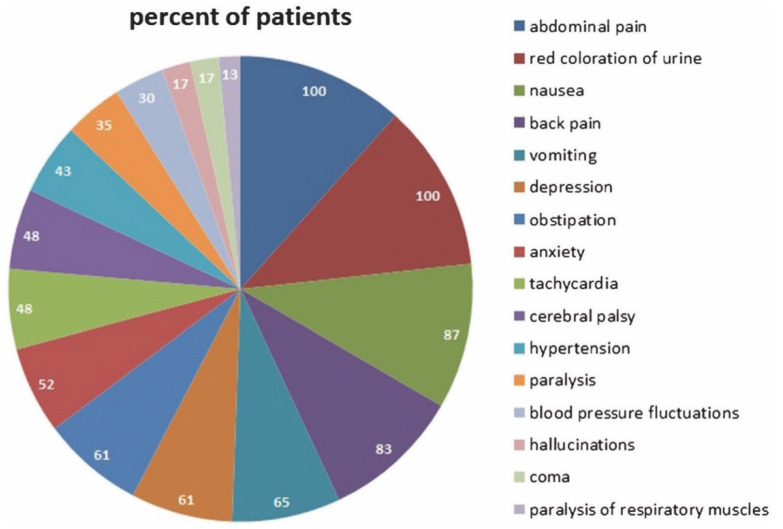
Signs and symptoms (in Percentages) during acute attacks in a German cohort of 62 patients (57 AIP, 5 VP) with acute porphyrias [16].

Attacks are characterised by a number of symptoms, the most prominent being abdominal pain. But also others, such as nausea, tachycardia and severe neurological complications (from altered sensation to life threatening paralysis) may occur (Figure 4). Many severely ill patients also develop hyponatremia, which contributes to the neurological symptoms [15].

Since 5-ALA and PBG strongly increase in urine during attacks, it is generally assumed that these heme precursors, in particular 5-ALA, cause the symptoms mentioned above (for a detailed discussion see [17]) through interaction with neurological structures such a GABA-receptors. However, although infusion of 5-ALA in a male volunteer led to an increase of 5-ALA and PBG to levels seen during acute attacks, this did not cause subjective symptoms in the individual [18]. Similar results have been obtained in mice [19]. Moreover, oral 5-ALA ingestion (Levulan^®^) has been approved in many countries for proto-porphyrin fluorescence in neurooncology without problems [20]. In addition, 5-ALA reduces glucose levels in mildly hyperglycermic subjects [21]. Moreover, oral intake of 5-aminolevulinic phosphate (alone or in combination with sodium ferrous citrate) has been studied in diabetics [22] and for the treatment of depression [23]. Hence, in Japan, 5-ALA is commercially available as a food supplement.

Moreover, in porphyria some gene carriers show high excretion of 5-ALA and PBG (so-called “high excreters”) without becoming symptomatic.

This indicates that 5-ALA elevation is necessary but not sufficient for the development of an acute attack. Possibly, high 5-ALA levels form products with other substances (called “unknown derivatives” by Bonkovsky in 1971) [24]. These postulated substances are elevated during the attack and eventually cause the neurological dysfunctions. This would be similar to the production of succinylacetone in hereditary tyrosinemia. Moreover, 5-ALA itself can be oxidized to 4,5-dioxovaleric acid (DOVA), which can alkylate DNA guanine moieties, promote protein cross linking and damage GABAergic receptors [25,26].

### 1.6. Natural History of the Disease: Severe and Non-Severe Patients, the “Chronic” Patient

In the majority of patients, acute porphyria is diagnosed in the hospital when the symptoms after remaining unrecognized for years have become so severe that treatment in the intensive care unit becomes necessary. If diagnosed accurately and treated with heme arginate (see below), many patients do not suffer from another attack in their life. The EPNET patients follow up guidelines group these individuals into group A (www.porphyria-europe.com Accessed on 18 January 2022). Some patients experience one or more additional attacks despite of change of life style (such avoidance of porphyrinogenic drugs, alcohol, nicotine). Since these attacks are anticipated, they are in general non-severe and can be immediately treated when prodromal symptoms become apparent (see below).

A third group, however, requires our special attention: these are mostly young females between 20 and 40 years of age. They suffer from serious impairment of their quality of life [15,27,28] since, until recently, no efficacious treatment was available (see below). These have usually **more than four recurrent attacks per year** and require heme arginate prophylactic treatment (the “chronic” patient). Although they avoid the major precipitating factors, the cause of the recurrence remains unidentified (termed **AIPr** for recurrent). It is likely that they harbour additional genetic alterations which favour the recurrent attacks.

### 1.7. Translational Projects

Many questions remain unanswered and should be addressed in future research: to answer these questions the dogma of a singular cause–effect has to be abandoned and replaced by a multiomic system biology approach which includes metabolomic, genomic, transcriptomic, lipidomic, gastronomic and proteomic levels [29]. In particular, it is unclear to what extent metabolic pathways other than homocysteine and tryptophan metabolism are altered by the dysregulation of porphyrin biosynthesis. It is still unclear which substance(s) cause the neurological symptoms. An untargeted metabolomics approach for their identification would be useful. If it were possible to identify the target structures of such neurotoxic substances, it may be possible to develop new types of antagonists for the treatment of acute attacks. It is also unknown how and why hyponatremia develops in some patients. Moreover, we do not know why some individuals (primarily women) suffer from recurrent attacks. Only one whole exome sequencing has been carried out in porphyria patients thus far, however [30]. Last but not least, it is not known which genetic and environmental factors contribute to the different penetrance of the acute porphyrias from individual to individual [31].

## 2. Therapy

### 2.1. General Aspects and Rationale

Always, the first measure is to identify factors such as certain medications which precipitate acute attacks and discontinue taking them.

Next, several pharmacological measures have become available over the last five decades, which will be discussed in detail. They all aim to reverse the dramatic metabolic dysregulation which leads to acute attacks.

### 2.2. Glucose Therapy-Pros and Cons

#### 2.2.1. Relationship of Heme and Energy Metabolism

Taking into consideration the intimate relationship between heme biosynthesis and intermediary metabolism (Figure 3, see above), it is not surprising that nearly 60 years ago it was already shown that diet can influence the excretion of porphyrin precursors, which usually fluctuate in patients with acute porphyrias [32]: a constant diet adequate in content of protein, fat and carbohydrate to maintain weight can diminish the excretion of the precursors, whereas reduction of the caloric intake was associated with a rise of 5-ALA and PBG. This effect was mainly due to the protein and carbohydrate content rather than the total caloric content of the diet.

In a study from Norway in 2018, it was found that increasing total energy intake was correlated with decreasing biochemical disease activity [33]: the intake of sugar and candies was higher in individuals with lower urine 5-ALA levels. Plasma insulin level was lower in those with high PBG levels, whereas hyperinsulinemia was observed in decompensated acute porphyria [34]. This was in line with observations that diabetes mellitus may have beneficial effects for patients with acute porphyrias [35,36,37] and experimental animals [38]. From these observations, the “glucose effect” was postulated: when high carbohydrate infusions (approximately up to 500 g/24 h) mainly in the form of glucose infusions were given, consistent and significant decreases of porphyrin precursors accompanied by clinical improvement in most of the patients were observed [39]. Others, however, have found that glucose therapy was not sufficient to achieve clinical and biochemical remission in more serious attacks [40]. This may be due to the fact that the Swedish authors used 200 instead 500 g glucose per day. Other investigators have applied glucose amounts from 450 to 600 g per day [24].

The effect of carbohydrates does not seem to be limited to glucose since intravenous laevulose (fructose) [41] and oral glycerol [42] has also been shown to be active.

**Figure 5:**
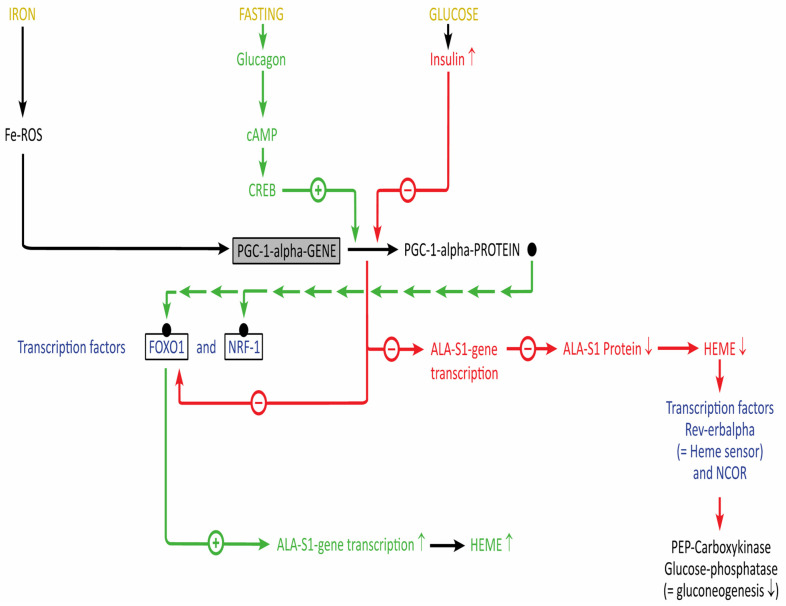
PGC-1-Alpha as a meeting point for the regulation of heme biosynthesis by fasting, glucose and iron (see text for details). Arrow up = increase, arrow down = decrease.

At present, the “glucose effect” is thought to be mediated by Coactivator 1 alpha (C-1alpha) of *P*eroxisome *P*roliferator *A*ctivated *R*eceptor Gamma (PPAR-gamma) which activates nuclear receptors and other transcription factors such as FOXO1 or NRF1 (Figure 5) (for a detailed discussion see also [43]). Fasting and glucagon upregulate P(PAR)G(amma)-C1 alpha [44], which is a key regulator of glucose and lipid metabolism, as well as circadian rhythms [45]: the hepatic 5-ALAS1 gene is upregulated by the interaction of the complexes formed by PGC-1 alpha, which couples with FOXO1 and nuclear respiratory factor 1 (NRF1) and a sequence of the 5-ALAS-1 promotor [46,47]. On the contrary, glucose stimulates insulin secretion, which antagonizes the induction of PGC-1 alpha expression and disrupts the interaction of PGC-1 alpha with FOXO1. To make things even more complicated, iron is also part of this regulatory process by PGC-1 alpha: it activates the transcriptional coactivator, which promotes heme biosynthesis. Heme binds to RevErbalpha (see Table 1), which leads to downregulation of hepatic glucose production [48,49].

In a Spanish study, 18% of the patients investigated showed a high HOMA-(Homeostasis Model Assessment)-IR (insulin resistance) index, which is used for the determination of the endogenous insulin resistance [50]. This index is calculated from glucose and insulin levels after a 12 h fast. Interestingly, patients with insulin resistance and hyperinsulinemia showed clinically stable porphyria disease. The nature of the insulin resistance in patients with AIP remains unknown.

Several additional studies addressed metabolic aspects in acute porphyria: for instance, after glucose loading pyruvate and lactate levels were significantly higher in patients with AIP in clinical remission than in controls [51]. When analysed for serum proteins, it was found that insulin-like growth factor I (IGF-1) and transerythrin (=prealbumin) were lower in chronic AIP patients (*n* = 14) who had more than one attack in less than four years than in patients (*n* = 12) who presented with a single acute attack and remained asymptomatic thereafter [52].

In a rat model, porphyrinogenic drugs altered the status of hormones that regulate glucose metabolism by increasing insulin and decreasing glucocorticoid levels [53]. In a mouse model, wild type and AIP mice (PBG-deaminase activity inhibited by genetic manipulation) respond differently to a 14 h fasting period: the healthy mice stimulate glycogen degradation to maintain glucose homeostasis, while the sick mice activate gluconeogenesis and ketogenesis since they are not able to use stored glycogen [54]).

In order to better understand the metabolic derangements in acute porphyria, first metabolomic studies have been carried out: fingerprinting by ^1^H NMR spectroscopy of urine showed that glycine (the precursor of 5-ALA) increases in patients with frequent recurrences [55]. In another approach, urinary metabolic profiling of asymptomatic porphyria gene carriers using again NMR spectroscopy found higher levels of acetate, citrate or pyruvate when compared to patients with porphyria cutanea tarda [56].

In serum of AIP patients, the levels of tyrosine and valine determined by LC/MS/MS were lower, whereas those of sarcosine (N-methylglycine), ornithine, and citrulline were significantly higher [57]. Sarcosine is part of the metabolism of glycine; ornithine and citrulline are important metabolites of the urea cycle.

Since heme biosynthesis partly takes place in the mitochondrium, studies have been initiated to study mitochondrial bioenergetics in peripheral blood monocytes in patients in acute porphyrias [58]. As a result, patients with moderate/severe symptoms, had significantly lower oxygen consumption rates than those with no or mild symptoms indicating a deficiency of mitochondrial function possibly due to limitations of electron transport and ATP production. Similar results were observed when using the XF96 analyzer for mitochondrial respiration [59].

In an experimental model (silencing PBG-deaminase), it could be shown that alpha-lipoic acid, which is a cofactor of alpha-ketoglutarate dehydrogenase in the TCA cycle i.e., the enzyme, which provides succinyl-CoA for heme biosynthesis, ameliorated heme biosynthesis and improved ATP production [60]. Since alpha-lipoic acid is commercially available as a nutritional supplement, a clinical study evaluating its potential effect may be worthwhile.

When patients were analysed not only for insulin and C-peptide, but also for cytokines, all cytokines measured were increased in symptomatic gene carriers as compared to healthy controls: in particular, high levels were found for visfatin (an adipokine with insulin-like activity), or interleukin 17, suggesting a T-helper type 17 inflammatory response [61].

Moreover, impairment of heme biosynthesis through experimental knockdown of 5-ALA-Synthase 1 (equivalent to siRNA silencing, see below) in mice causes impaired glucose tolerance and insulin resistance [62].

These observations have established carbohydrate loading as the **first line therapeutic approach** to downregulate hepatic 5-ALAS1 transcription. However, the response of different patients to carbohydrate loading ranges from spectacular over little to no effect. This may in part be due to different glucose dosages used (300 to 500 g, with or without insulin). Glucose infusion is particularly valuable since the sugar is readily available and inexpensive and heme arginate (see below) usually has to be ordered from the pharmaceutical provider, which may take one or two days to arrive. Therefore, carbohydrate loading is useful as a bridging therapy (pro). In patients with hyponatremia caution is, however, necessary since glucose infusion may aggravate hyponatremia [63]. Since the effect is not strong and long lasting enough to counteract severe (in particular neurological) complications of porphyric attacks (con) more efficacious therapies had to be developed: in an experimental mouse model, glucose was coadministered with a targeted insulin. Normally insulin acts on several organs such as liver, muscle or adipose tissue. Chemical coupling of insulin to apolipoprotein A-1 targets insulin preferentially into the hepatocyte and enhances the effect of glucose therapy [50].

#### 2.2.2. Translational Projects

The best option, of course, would be to replace the missing enzyme using enzyme replacement therapy (see below). As long as this therapy is not available, a better understanding of the metabolic consequences of the altered energy metabolism in acute porphyrias and their reprogramming may improve the efficacy of the therapeutical approach. Novel approaches, such as targeted and untargeted metabolomics as well as proteomics, will be helpful. In addition, a closer cooperation between porphyrinologists and endocrinologists would be beneficial.

### 2.3. Heme/Hemin Therapy-Pro and Cons

#### 2.3.1. Heme/Hemin for Treatment of Acute Attacks

Providing heme to a patient with an acute attack makes sense because it will inhibit 5-ALAS-1 activity while simultaneously replenishing the heme pool.

Hemin (Panhematin^®^) was approved in the US in **1983**, heme arginate (Normosang^®^) in **2006** in the European Union. One lot of Panhematin^®^ contains 350 mg of lyophilized hemin black powder, whereas one 10 mL vial of Normosang^®^ contains 250 mg human hemin plus arginine (as counter ion) plus ethanol (as a solvent) (Table 2). The alcohol content corresponds to 24 mL beer or 10 mL wine per ampoule. 250 mg heme contain 23 mg iron (see below). Since heme arginate is barely soluble in aequous solutions like blood plasma, the solution is mixed with 10 mL 20% human serum albumin (=2 g albumin per vial), which acts as a transient heme deposit prior to infusion. Others [64,65] use higher albumin concentrations (33 g per vial hematin). Both preparations, Normosang^®^ and Panhematin^®^, seem to have different stabilities [66]. When repeated infusions are necessary, the best access is through a totally implantable venous access port that will avoid the risk of phlebitis of a peripheral vein.

Meanwhile, hemin/heme has been established as the **standard of care** for the treatment of acute attacks.

After intravenous infusion, heme is transferred from albumin to hemopexin, which is then taken up as heme–hemopexin complex by hepatic parenchymal cells via receptor mediated endocytosis. One has to keep in mind that the heme amount present in one vial of Normosang^®^ (250 mg) is five times the amount produced by the liver per day.

Prior to approval and commercial distribution, several case reports (in chronological order) of patients with acute porphyrias have been published: the first by Bonkovsky et al. in 1971 [24] in the US (NIH, Bethesda) who used **hemin** produced from packed red blood cells in a female patient with severe AIP attacks. Since she was refractory to all prior treatments, hemin was tried: although there was a clear biochemical improvement (suppression of the production of precursors) the patient deceased with renal failure because of the severity of the disease. This was, however, not the first intravenous administration of hematin, which had been given to 11 volunteers 26 years earlier to study the conversion of hematin to bilirubin [67].

The NIH group reported four years later in three AIP and one variegate porphyria (VP) patient not only biochemical but also “prompt and gratifying” clinical improvement [68]. In 1976 the same group [69] described the complete neurological recovery in a female patient upon hematin infusion. In 1977 Watson et al. (Minneapolis) reported having treated 20 patients (13 AIP, 6 VP, 1 hereditary copropoprhyria (HCP)) with 31 attacks with hematin solution prepared in the laboratory: 25 of the attacks responded well [70,71]. Lamon et al. [72] treated in 1979 12 patients with hematin and measured intravascular hematin clearance and hemopexin as well as urine 5-ALA and PBG. They observed a chemical decrease of urine PBG and 5-ALA and clinical improvement (abdominal and other pain, weakness, but not on paresis or paralysis).

In 1981 McColl et al. [73] published the results of 8 patients with 13 attacks: they also showed biochemical as well as clinical response. Finally, Pierach et al. in 1980 and Pierach in 1982 (Minneapolis) gave a general overview of his experience with hematin [74,75], having treated 57 patients (of these 43 with AIP) treated. Of these, 90% showed clinical response. In 2007 Kuo et al., (Taiwan) described the improvement of neurological complications upon hematin infusions [76]. In one patient in New York hemin treatment was combined with hemodialysis [77].

In 1986 Mustajoki et al., in Finland reported on **heme arginate**: six patients with AIP and four with VP [78]. In 1987 Herrick et al. in Scotland [79] treated seven attacks in five patients: decrease of 5-ALA and PBG and improvement of clearance of antipyrin (which is degraded in the liver by CYP enzymes) were observed. In 1988 Tokola et al., (Finland) reported on six patients with VP with respect to antipyrin clearance [80]. In 1988 Volin et al. [81] did not find adverse effects on hemostasis in healthy volunteers treated with heme arginate.

In 1989 Herrick et al. [82] reported the **only double blind study** comparing placebo and heme arginate in 12 patients: urine precursors were decreased but clinical response (indices of severity: analgesic requirement, pain score, duration of hospitalization) was less striking. In 1991 Kostrzewska et al., in Poland treated 47 attacks of AIP patients: the best clinical responses were obtained with early treatment and in patients without neurological symptoms [83].

In 1993 Mustajoki (Finland) and Yves Nordmann (France) [84] treated 22 patients with AIP and 2 patients with VP: all patients were responders (abdominal pain, opiate discontinuation, hospital time). The only side effect was thrombophlebitis in one individual. In the same year Muthane et al. [85] found glucose infusions not to be effective enough but heme arginate.

In 2011 Ma et al. [86] showed that heme arginate is also active in patients with HCP; in 2020 efficacy was also reported in Galveston for patients with 5-ALA-dehydratase porphyria [87]. In rare cases, hypersensitivity to heme arginate has been observed: in such a situation, heme arginate can be given again when the drug is applied in a slowly escalating dose (such as rituximab in the treatment of lymphomas) [88].

In some patients, loss of activity of heme arginate upon repeated infusions was observed: since it has been postulated that the induction of heme oxygenase is responsible, Dover et al. [89] claimed that inhibition of heme oxygenase by tin protoporphyrin would prolong the biochemical remission induced by heme arginate in acute porphyrias. However, this has not been proven by other investigators. Moreover, the substance is difficult to obtain on the market.

In pregnant women Isenschmidt et al. [90] recommended high dose glucose therapy and, if not effective, heme therapy.

In summary: although the number of published studies describing clinical and chemical effects of the two heme preparations is low and only one randomized clinical trial has been carried out, there is convincing evidence to advocate the recommendation that intravenous heme should be used as a first line treatment especially in patients with severe attacks. This recommendation is further supported by unpublished real world experience with the drugs. As a bridging therapy (i.e., until the drug is delivered to the treating unit) or for patients with milder attacks glucose infusions still retain their value.

#### 2.3.2. Prophylactic Heme/Hemin Therapy

Although not approved for prophylactic therapy, heme or hematin preparations, respectively, are used in many countries to prevent recurrent attacks. Due to the lack of approval, there are no published guidelines for prophylactic use of heme.

Kuo et al. in Taiwan [91] reported on five patients who received weekly heme arginate infusions over a period of up to 15 years. Under the weekly infusion of one vial of heme, the yearly attack rate was reduced from 11.82 to 2.23. There were no adverse on liver and renal function observed; the only problem was port infections. Yarra et al. in Winston-Salem [92] reported on two patients treated weekly with one vial for up to one year. They observed much better symptom control. Interestingly, the urine precursors 5-ALA and PBG remained elevated, although the frequency of the attacks was decreased. Possibly, precursor levels went down and reincreased again on the day of the next infusion.

In 2006 Anderson (Galveston) and Collins (Ovation Pharmaceuticals) in the US reported in an open label study on the use of hemin in 111 patients with 305 acute attacks [93]: of these 40 patients with recurrent attacks were treated prophylactically with either one vial weekly or every two weeks. In addition, various other regimens were used: three times a week, twice a week, every two or four weeks, daily infusions over three days every six weeks, twice daily infusions every one to two weeks, etc. Moreover, the regimen varied in some patients over the course of the study, reflecting the empirical approach. Side effects were headache, nausea, phlebitis, catheter complications, pain and skin reactions. The researchers conclude that “prophylactic use of hemin (Panhematin^®^) is common (31% of patients) and is perceived as effective in up to 68% of patients”. They add that there is little information how hemin should be used in clinical practice.

In 2014 Bonkovsky et al. [94] described 108 patients from the US Porphyria consortium of whom 26% repeatedly received hematin infusions.

In 2015 Marsden et al. in England reported [95] on 22 patients (21 females, 1 male) who received regular heme arginate infusions to prevent recurrent symptoms. Again, the treatment schedule was quite variable: four patients received one dose (=3 mg/kg/day), three patients two doses, one patient six and another eight doses per month. According to the authors, no patients developed clinical signs of iron overload. However, the majority of the patients had strongly elevated ferritin levels of up to 3165 ng/mL and no quantitative non-invasive liver iron measurements by T2*MRI were reported. The authors conclude that regular heme arginate infusions coincided with clinical improvement in 50–70% of their patients.

Schmitt et al., in France [96] investigated 46 patients with recurrent attacks under heme arginate treatment, four vials of heme arginate contain 92 mg iron (total body iron content in human beings on average 4000 mg). Using quantitative liver iron determination by T2* MRI they found mild to severe iron overload in 11/11 patients investigated. The authors conclude that chronic heme infusions lead to a chronic inflammatory stage in the liver because of an induction of heme oxygenase 1.

Willandt et al. in Belgium and the Netherlands [97] report that regular heme arginate application causes not only an increase of serum ferritin but also promotes liver fibrosis.

#### 2.3.3. Economical Aspects of Prophylactic Heme Therapy

Connolly et al. [98] and Neeleman et al. [99] in the Netherlands investigated AIP patients with recurrent attacks: both groups found a high morbidity compared to other symptomatic patients. Recurrent attacks resulted in high medical costs and unemployment rates.

Blaylock et al. in the US [100] compared the expenditures for patients treated repeatedly for acute attacks vs. prophylactically and came to the conclusion that costs are significantly lower for patients treated prophylactically (see also Section 2.4.2).

In summary, although only one randomized clinical study is available, real world data show that the use of heme is beneficial when patients suffer from acute attacks. However, the minimal effective dose and the optimal regimen have still not been established. An oral heme preparation that can be absorbed by the gut would be highly desirable as stated by Bonkovsky et al. some 40 years ago [101]. In addition, other phenomena, such as oscillations of hepatic 5-ALA-Synthase, have not been taken into consideration [102].

When used prophylactically, treatment has to be individualized but the optimal strategy with treatment criteria is not known yet.

#### 2.3.4. Translational Projects

Iron overload is a concern with heme treatment: when patients are treated prophylactically, an early basic quantitative liver iron measurement by T2*MRI should be carried out and used for follow up since ferritin determinations are not fully reliable (ferritin is also an acute phase reactant) and can fluctuate drastically in AIP patients. An increase of the liver iron content should be early counteracted with iron removal measures.

### 2.4. Givosiran Therapy-Pros and Cons

#### 2.4.1. Givosiran Therapy in Acute Porphyrias

Givosiran (generic name) therapy based upon siRNA (small interfering) technology has been a revolutionary introduction into the field: this therapeutical system has been developed by the group of Thomas Tuchel in Göttingen (Max Planck Institute) and patented [103]. With siRNAs, the transcription of certain mRNAs can be blocked in a target cell. If the first enzyme of porphyrin biosynthesis, 5-ALA synthase 1, could be partially inhibited in the hepatocyte, this would lead to an inhibition of the flooding of the organism with 5-ALA and PBG during decompensation of heme biosynthesis during acute attacks.

Synthetic double stranded DNA that contains an 5-ALAS1 specific sequence is derivatised with N-acetylgalactosamine to target the asialoglyoprotein receptor (ASGP-R), which is expressed nearly exclusively on hepatocytes. In the hepatocyte the RNA is processed into approximately 20 bp fragments by a cellular enzyme and then separated into single strands. The complementary strand binds to cellular ALAS1 mRNA. The newly formed double stranded RNA is then degraded.

Initially givosiran was tested in a mouse model where it caused a dose dependent inhibition of 5-ALAS-1 [104].

To search for possible interactions of this inhibition with the activity of drug metabolizing CYP enzymes, five of these were tested in subjects with AIP who were chronic porphyrin precursor excreters: no major impact on activity could be observed [105].

After phase 1/2 studies, the substance was tested in the phase 3 ENVISION-study (givosiran vs. Placebo for 6 months and subsequent verum application): 6 month [106] and 24 month analyses [107] showed a convincing efficacy of the drug: patients with recurrent attacks showed reduced attack frequency, reduced hemin use and less daily pain while improving quality of life.

However, with regard to safety there is some major concern: in addition to chronic kidney disease [108] severe adverse effects such as cerebral venous thrombosis, pulmonary embolism and pancreatitis were observed. At least in part these serious adverse events could be explained by the excessive elevations of homocysteine (up to 400 µmol/L), which we had already observed in 2020 and published in 2021 based on our two Envision patients [109]. By now this observation has been confirmed in the majority of givosiran-treated patients by several international groups [110,111,112,113] and investigations of the sponsoring company Alnylam on frozen study patient samples. As mentioned earlier, in most patients with active porphyria patients there is already a mild disturbance of homocysteine metabolism.

Meanwhile, several dosing attempts have been made to cope with the problem through vitamin supplementation. At present the optimal strategy is debated by an international group of experts: currently 80 mg vitamin B6 per day seems to be the best available regimen [114].

Dosing of the drug givosiran (Givlaari^®^) is recommended as a monthly application. We have observed that homocysteine only slowly declines after discontinuation of givosiran: this indicated an effect of the drug exceeding the normal four week period [109]. A recent publication confirmed the sustained efficacy of givosiran in most patients: this allowed the authors to personalize dosing frequency [115] using lack of symptoms and low urinary heme precursor levels as the criteria for individualization. With this strategy two subgroups were defined: patients with a moderate or unstable decrease in 5-ALA level (58%) who required injections every three months or less, and patients with prolonged and stable low 5-ALA levels (42%) who were only treated when heme precursor levels in urine were increasing again. Two of the patients required dosing only after 8 or 11 months, respectively, after the previous one. The authors also confirmed a high prevalence of adverse events: one patient discontinued treatment due to acute pancreatitis, another due to pulmonary embolism. All patients had hyperhomocysteinemia, and all patients with initial homocysteine measurements available showed an increase under treatment. The authors conclude that the sustained effect of givosiran allowed a decrease in dosing frequency without compromising treatment efficacy. The high prevalence of adverse events emphasizes the importance of restricting the treatment to severe AIP and administering the minimum effective dose for each patient.

The observations of the authors also suggest that givosiran is most effective when given early in the disease course.

Interestingly, two of the patients who responded less to givosiran had a c.849G—A HMBS mutation: this may indicate that the mutated mRNA somehow interacts with the siRNA. Alternatively, autoantibody formation against the asialoglycoprotein receptor may impair the uptake of the siRNA [116].

Nonetheless, inhibition of 5-ALS1 is very efficacious in reducing the number of recurrent attacks in AIP patients. However, the fact that this therapy acts through partial inhibition of a metabolic pathway may have unwanted impact on other metabolic pathways.

In the future, a **therapeutic algorithm** needs to be developed that incorporates heme as well as givosiran treatments.

#### 2.4.2. Economical Aspects

Massachi et al. [117] compared the costs for givosiran with those for hemin in the US and arrived at the conclusion of lower annual total costs of care with hemin treatment. The novel individualized dosing approach (see above) had, however, not been available at the time of this analysis.

In a future analysis, the drug agency refund for these drugs in various parts of the world should be assessed.

#### 2.4.3. Translational Projects

Since experimental inhibition of 5-ALA-Synthase 1 alters the HOMA-index in experimental animals (see above), it would be interesting to measure this index in patients on givosiran treatment. Moreover, other CYP enzyme pathways such as those involved in testosterone degradation should be analyzed during givosiran therapy.

## 3. New Therapeutical Approaches

For better treatment of patients with acute porphyrias, enzyme substitution therapy would be ideal. This therapy has been shown for a long time to be efficacious and safe in other metabolic disorders such as Gaucher disease. For this reason, this approach was also investigated in acute intermittent porphyria: since directing the recombinant enzyme Porphozym^®^ (Zymenex Corporation, Hillerod, Denmark) into the hepatocyte was not successful in a clinical study, this approach unfortunately had been given up [118]. Meanwhile, however, a novel approach coupling recombinant PBG-deaminase with apolipoprotein A1 and subsequent intravenous or subcutaneous injection led to a long-lasting therapeutic effect [119].

Alternative therapeutical strategies are also being developed: in animal models the efficacy of a mRNA, which codes for PBG-deaminase, was demonstrated. Injection of this mRNA led—following the same principle as used for the COVID-19 vaccines developed by BioNtech and Moderna—to a reconstruction of the PBG-deaminase activity so that mice and primates can survive a drug stress without a problem [120]. Another approach tries to slow down the degradation of the non-mutated enzyme so that the total enzyme activity increases [121]. We can therefore expect that, in the coming years, interesting novel therapeutical options will become available.

## 4. Conclusions

The field of molecular understanding of acute porphyrias and the treatment of patients with these diseases has reached a fascinating phase: established therapies such as carbohydrate loading and heme therapy are being investigated in more detail to optimize their efficacy. Innovative therapies such as siRNA approaches are being further developed to reach an acceptable risk benefit ratio. Moreover, other novel approaches are on the way into the clinic for the benefit of our patients.

## Figures and Tables

**Table 1:** Functions of heme in human cellular metabolism.

Heme Protein	Function
Cytochrome c	electron transport
Hemoglobin	oxygen transport
Myo- und Neuroglobin	oxygen storage
Cytochrome P450	metabolism of drugs and steroids
5-ALA-Synthase 1	regulation of enzyme activity
Transcription factor BACH 1	regulation of heme oxygenase
ferritin and ferroportin	
Cystathionine-ß-Synthase	degradation of homocysteine
Myeloperoxidase Nitric oxid synthases	formation of HOCL in neutrophils production of NO from arginine
Rev-Erb-alpha/ß	transcription factor (circadian rhythm)
K+-channels	ion transporters

**Table 2:** Panhematin^®^ (US) and Normosang^®^ (Europe) Preparations in 2022 (both manufactured and distributed by Recordati Rare Diseases Inc., Milan, Italy).

	Panhematin^®^	Normosang^®^
Preparation	lyophilized powder	10 mL concentrated solution
Heme content per vial	350 mg	250 mg
Additional ingredients	240 mg sodium carbonate 335 mg sorbitol	propylenglykol 96% ethanol (1 g/10 mL)
		Arginine
Storage temperature	20–25 °C	2–8 °C
Maximum Duration of stability		2 years
Recommended dosage	1–4 mg/kg/day for 4 days	3 mg/kg/day for 3–14 days
Use of sterile filter/glass bottle recommended	yes/yes	yes/no

## Data Availability

No applicable.

## References

[1] Zhang L. (2020). Heme Biology: Heme Acts as a Versatile Signaling Molecule Regulating Diverse Biological Processes.

[2] Shimizu T., Lengalova A., Martínek V., Martínková M. (2019). Heme: Emergent roles of heme in signal transduction, functional regulation and as catalytic centres. Chem. Soc. Rev..

[3] Kühl T., Imhof D. (2014). Regulatory Fe II/III Heme: The reconstruction of a molecule´s biography. ChemBioChem.

[4] Taoka S., Lepore B.W., Kabil O., Ojha S., Ringe D., Banerjee R. (2002). Human cystathionine beta-synthase is a heme sensor protein. Evidence that the redox sensor is heme and not the vicinal cysteines in the CXXC motif seen in the crystal structure of the truncated enzyme. Biochemistry.

[5] Tolosano E., Fagoonee S., Morello N., Vinchi F., Fiorito V. (2010). Heme scavenging and the other facets of hemopexin. Antioxid. Redox Signal..

[6] Owen O.E., Kalhan S.C., Hanson R.W. (2002). The key role of anaplerosis and cataplerosis for citric acid cycle function. J. Biol. Chem..

[7] Ryter S.W. (2021). Heme Oxgenase-1, a Cardinal Modulator of Regulated Cell Death and Inflammation. Cells.

[8] Yoshida Y., Ashino T., Kobayashi Y. (2016). Chemical-induced and reciprocal changes in heme metabolism, cytochrome P450 synthesis and others in the liver of human and rodents. J. Toxicol. Sci..

[9] Philipps J.D. (2019). Heme biosynthesis and the porphyrias. Mol. Gen. Metab..

[10] Hahn D., Shin S.H., Bae J.S. (2020). Natural Antioxidant and Anti-Inflammatory Compounds in Foodstuff or Medicinal Herbs Inducing Heme Oxygenase-1 Expression. Antioxidants.

[11] Yasuda M., Chen B., Desnick R.J. (2019). Recent advances on Porphyria genetics: Penetrance & molecular heterogeneity, including new modifying/causative genes. Mol. Gen. Metab..

[12] To-Figueras J., Lopez R.M., Deulofeu R., Herrero C. (2010). Preliminary report: Hyperhomocysteinemia in patients with acute intermittent porphyria. Metabolism.

[13] Ventura P., Corradini E., Di Pierro E., Marchini S., Marcacci M., Cuoghi C., Buzzetti E., Pietrangelo A. (2020). Hyperhomocysteinemia in patients with acute porphyrias: A potentially dangerous metabolic crossroad?. Eur. J. Intern. Med..

[14] Gomez-Gomez A., Aguilera P., Langohr K., Casals G., Pavon C., Marcos J., To-Figueras J., Pozo O.J. (2022). Evaluation of Metabolic Changes in Acute Intermittent Porphyria Patients by Targeted Metabolomics. Int. J. Mol. Sci..

[15] Bronisch O., Stauch T., Haverkamp T., Beykirch M.K., Petrides P.E. (2019). Acute porphyrias: A German monocentric study of the biochemical, molecular genetic, and clinical data of 62 families. Ann. Hematol..

[16] Zainuddin N.M., Sthnashwar P., DB Vethakkan S.R. (2019). Acute intermittent porphyria: A rare cause of hyponatremia. Malays. J. Pathol..

[17] Ricci A., Di Pierro E., Marcacci M., Ventura P. (2021). Mechanisms of Neuronal Damage in Acute Hepatic Porphyrias. Diagnostics.

[18] Mustajoki P., Timonen K., Gorchein A., Seppäläinen A.M., Matikainen E., Tenhunen R. (1992). Sustained high plasma 5-aminolaevulinic acid concentration in a volunteer: No porphyric symptoms. Eur. J. Clin. Investig..

[19] Edwards S., Jackson D., Reynoldson J., Shanley B. (1984). Neuropharmacology of δ-aminolaevulinic acid. II. Effect of chronic administration in mice. Neurosci. Lett..

[20] Cozzens J.W., Lokaitis B.C., Moore B.E., Amin D.V., Espinosa J.A., MacGregor M., Michael A.P., Jones B.A. (2017). A Phase 1 Dose-Escalation Study of Oral 5-Aminolevulinic Acid in Adult Patients Undergoing Resection of a Newly Diagnosed or Recurrent High-Grade Glioma. Neurosurgery.

[21] Higashikawa F., Noda M., Awaya T., Tanaka T., Sugiyama M. (2013). 5-aminolevulinic acid, a precursor of heme, reduces both fasting and postprandial glucose levels in mildly hyperglycemic subjects. Nutrition.

[22] Nakamura Y., Haraguchi A., Shigeno R., Ito A., Horie I., Kawakami A., Abiru N. (2021). A single-arm, open-label, intervention study to investigate the improvement of glucose tolerance after administration of the 5-aminolevulinic acid (5-ALA) in the patients with mitochondrial diabetes mellitus. Medicine.

[23] Higashikawa F., Kanno K., Ogata A., Sugiyama M. (2020). Reduction of fatigue and anger-hostility by the oral administration of 5-aminolevulinic acid phosphate: A randomized, double-blind, placebo-controlled, parallel study. Sci. Rep..

[24] Bonkowsky H.L., Tschudy D.P., Collins A., Doherty J., Bossenmaier I., Cardinal R., Watson C.J. (1971). Repression of the overproduction of porphyrin precursors in acute intermittent porphyria by intravenous infusions of hematin. Proc. Natl. Acad. Sci. USA.

[25] Bechara E.J.H., Ramos L.D., Stevani C.V. (2021). 5-Aminolevulinic acid: A matter of life and caveats. J. Photochem. Photobiol..

[26] Bechara E.J.H., Dutra F., Cardoso V.E.S., Sartori A., Olympio K.P.K., Penatti C.A.A., Adhikari A., Assunção N.A. (2007). The dual face of endogenous alpha-aminoketones: Pro-oxidizing metabolic weapons. Comp. Biochem. Physiol. Part C Toxicol. Pharmacol..

[27] Gouya L., Ventura P., Balwani M., Bissell D.M., Rees D.C., Stölzel U., Phillips J.D., Kauppinen R., Langendonk J.G., Desnick R.J. (2020). Explore: A Prospective, Multinational, Natural History Study of Patients with Acute Hepatic Porphyria with Recurrent Attacks. Hepatology.

[28] Naik H., Stoecker M., Sanderson S.C., Balwani M., Desnick R.J. (2016). Experiences and concerns of patients with recurrent attacks of acute hepatic porphyria: A qualitative study. Mol. Genet. Metab..

[29] Karczewski K., Snyder M. (2018). Integrative omics for health and disease. Nat. Rev. Genet..

[30] Goncharova M., Pshenichnikova O., Luchinina Y., Pustovoit Y., Karpova I., Surin V. (2019). Molecular genetic study of acute intermittent porphyria in Russia: HMBS gene mutation spectrum and problem of penetrance. Clin. Genet..

[31] Lenglet H., Schmitt C., Grange T., Manceau H., Karboul N., Bouchet-Crivat F., Robreau A.M., Nicolas G., Lamoril J., Simonin S. (2018). From a dominant to an oligogenic model of inheritance with environmental modifiers in acute intermittent porphyria. Hum. Mol. Genet..

[32] Welland F.H., Hellman E.S., Gaddis E.M., Collins G., Hunter G.W., Tschudy D.P. (1964). Factors affecting the excretion of porphyrin precursors by patients with acute intermittent porphyria. I. The effect of diet. Metabolism.

[33] Storjord E., Dahl J.A., Landsem A., Ludviksen J.K., Karlsen M.B., Karlsen B.O., Brekke O.L. (2019). Lifestyle factors including diet and biochemical biomarkers in acute intermittent porphyria: Results from a case-control study in northern Norway. Mol. Genet. Metab..

[34] Sixel-Dietrich F., Verspohl F., Doss M. (1985). Hyperinsul.linemia in acute intermittent porphyria. Horm. Metab. Res..

[35] Yalouris A.G., Raptis S.A. (1987). Effect of diabetes on porphyric attacks. BMJ.

[36] Lithner F. (2002). Beneficial Effect of Diabetes on Acute Intermittent Porphyria. Diabetes Care.

[37] Andersson C., Bylesjö I., Lithner F. (1999). Effects of diabetes mellitus on patients with acute intermittent porphyria. J. Intern. Med..

[38] Bitar M., Weiner M. (1984). Diabetes induced alterations in heme synthesis and degradation and various heme containing enzymes in female rats. Diabetes.

[39] Doss M., Sixel-Dietrich F., Verspohl F. (1981). “Glucose effect” and rate limiting function of uroporphyrinogen synthase on porphyrin metabolism in hepatocyte culture: Relationship with human acute hepatic porphyrias. Clin. Chem. Lab. Med..

[40] Sardh E., Harper P., Andersson D.E., Floderus Y. (2009). Plasma porphobilinogen as a sensitive biomarker to monitor the clinical and therapeutic course of acute intermittent porphyria attacks. Eur. J. Intern. Med..

[41] Brodie M.D., Moore M.R., Thompson G.G., Goldberg A. (1977). The treatment of acute intermittent porphyria with laevulose. Clin. Sci. Mol. Med..

[42] Bonkowsky H.L., Magnussen C.R., Collins A.R., Doherty J.M., Hess R.A., Tschudy D.P. (1976). Comparative effects of glycerol and dextrose on porphyrin precursor excretion in acute intermittent porphyria. Metabolism.

[43] Di Pierro E., Granata F. (2020). Nutrients and Porphyria: An intriguing crosstalk. Int. J. Mol. Sci..

[44] Liang H., Ward W.F. (2006). PGC-1alpha: A key regulator of energy metabolism. Adv. Physiol. Educ..

[45] Handschin C., Spiegelman B.M. (2006). Peroxisome proliferator-activated receptor gamma coactivator 1 coactivators, energy homeostasis, and metabolism. Endocrine. Rev..

[46] Handschin C., Lin J., Rhee J., Peyer A.K., Chin S., Wu P.H., Meyer U.A., Spiegelman B.M. (2005). Nutritional regulation of hepatic heme biosynthesis and porphyria through PGC-1alpha. Cell.

[47] Li D. (2005). PGC-1alpha: Looking behind the sweet treat for porphyria. Cell.

[48] Simcox J.A., Mitchell T.C., Gao Y., Just S.F., Cooksey R., Cox J., Ajioka R., Jones. D., Lee S.H., King D. (2015). Dietary iron controls circadian hepatic glucose metabolism through heme synthesis. Diabetes.

[49] Yin L., Wu N., Curtin J.C., Qatanani M., Szwergold N.R., Reid R.A., Waitt G.M., Parks D.J., Pearce K.H., Wisely G.B. (2007). Rev-erbalpha, a heme sensor that coordinates metabolic and circadian pathways. Science.

[50] Solares I., Izquierdo-Sánchez L., Morales-Conejo M., Jericó D., Castelbón F.J., Córdoba K.M., Sampedro A., Lumbreras C., Moreno-Aliaga M.J., Enríquez De Salamanca R. (2021). High Prevalence of Insulin Resistance in Asymptomatic Patients with Acute Intermittent Porphyria and Liver-Targeted Insulin as a Novel Therapeutic Approach. Biomedicines.

[51] Herrick A.L., Fisher B.M., Moore M.R., Cathcart S., Mccoll K.E., Goldberg A. (1990). Elevation of blood lactate and pyruvate levels in acute intermittent porphyria—A reflection of haem deficiency?. Clin. Chim. Acta.

[52] Delaby C., To-Figueras J., Deybach J.C., Casamitjana R., Puy H., Herrero C. (2009). Role of two nutritional hepatic markers (insulin-like growth factor 1 and transthyretin) in the clinical assessment and follow-up of acute intermittent porphyria patients. J. Intern. Med..

[53] Matkovic L.B., D’andrea F., Fornes D., San Martín De Viale L.C., Mazzetti M.B. (2011). How porphyrinogenic drugs modeling acute porphyria impair the hormonal status that regulates glucose metabolism. Their relevance in the onset of this disease. Toxicology.

[54] Collantes M., Serrano-Mendioroz I., Benito M., Molinet-Dronda F., Delgado M., Vinaixa M., Sampedro A., Enríquez de Salamanca R., Prieto E., Pozo M.A. (2016). Glucose metabolism during fasting is altered in experimental porphobilinogen deaminase deficiency. Hum. Mol. Genet..

[55] Carichon M., Pallet N., Schmitt C., Lefebvre T., Gouya L., Talbi N., Deybach J.C., Beaune P., Vasos P., Puy H. (2014). Urinary metabolic fingerprint of acute intermittent porphyria analyzed by (1)H NMR spectroscopy. Anal. Chem..

[56] Luck M., Schmitt C., Talbi N., Gouya L., Caradeuc C., Puy H., Bertho G., Pallet N. (2018). Urinary metabolic profiling of asymptomatic acute intermittent porphyria using a rule-mining-based algorithm. Metabolomics.

[57] Lin C., Shiao M., Cheng M., Chen C., Kuo H. (2021). Profiling of Serum Metabolites of Acute Intermittent Porphyria and Asymptomatic HMBS Mutation Carriers. Cells.

[58] Dixon N., Li T., Marion B., Faust D., Dozier S., Molina A., Rudnick S., Bonkovsky H.L. (2019). Pilot study of mitochondrial bioenergetics in subjects with acute porphyrias. Mol. Genet. Metab..

[59] Chacko B., Culp M.L., Bloomer J., Phillips J., Kuo Y., Darley-Usmar V., Singal A.K. (2019). Feasibility of cellular bioenergetics as a biomarker in porphyria patients. Mol. Genet. Metab. Rep..

[60] Longo M., Paolini E., Meroni M., Duca L., Motta I., Fracanzani A.L., Di PIierro E., Dongiovanni P. (2021). α-Lipoic Acid Improves Hepatic Metabolic Dysfunctions in Acute Intermittent Porphyria: A Proof-of-Concept Study. Diagnostics.

[61] Storjord E., Dahl J.A., Landsem A., Fure H., Ludviksen J.K., Goldbeck-Wood S., Karlsen B.O., Berg K.S., Mollnes T.E., Nielsen E.W. (2017). Systemic inflammation in acute intermittent porphyria: A case-control study. Clin. Exp. Immunol..

[62] Saitoh S., Okano S., Nohara H., Nakano H., Shirasawa N., Naito A., Yamamoto M., Kelly V.P., Takahashi K., Tanaka T. (2018). 5-aminolevulinic acid (ALA) deficiency causes impaired glucose tolerance and insulin resistance coincident with an attenuation of mitochondrial function in aged mice. PLoS ONE.

[63] Solares I., Tejedor M., Jerico D., Molares-Conejo M., de Salamanca R.E., Fontanellas A. (2020). Management of hyponatremia associated with porphyria-proposal for the use of tolvaptan. Ann. Transl. Med..

[64] Bonkovsky H.L., Healey J.F., Lourie A.N., Gerron G.G. (1991). Intravenous heme-albumin in acute intermittent porphyria: Evidence for repletion of hepatic hemoproteins and regulatory heme pools. Am. J. Gastroenterol..

[65] Anderson K.E., Bonkovsky H.L., Bloomer J.R., Shedlofsky S.I. (2006). Reconstitution of hematin for intravenous infusion. Ann. Intern. Med..

[66] Goetsch C.A., Bissell D.M. (1986). Instability of hematin used in the treatment of acute hepatic porphyria. N. Engl. J. Med..

[67] Pass I.J., Schwartz S., Watson C.J. (1945). The conversion of hematin to bilirubin following intravenous administration in human subjects. J. Clin. Investig..

[68] Dhar G.J., Bossenmaier I., Petryka Z.J., Cardinal R., Watson C.J. (1975). Effects of hematin in hepatic porphyria. Further studies. Ann. Intern. Med..

[69] Peterson A., Bossenmaier I., Cardinal R., Watson C.J. (1976). Hematin treatment of acute porphyria. Early remission of an almost fatal relapse. JAMA.

[70] Watson C.J., Pierach C.A., Bossenmaier I., Cardinal R. (1977). Postulated deficiency of hepatic heme and repair by hematin infusions in the “inducible” hepatic porphyrias. Proc. Natl. Acad. Sci. USA.

[71] Watson C.J., Pierach C.A., Bossenmaier I., Cardinal R. (1978). Use of hematin in the acute attack of the “inducible” hepatic prophyrias. Adv. Intern. Med..

[72] Lamon J.M., Frykholm B.C., Hess R.A., Tschudy D.P. (1979). Hematin therapy for acute porphyria. Medicine.

[73] McColl K.E., Moore M.R., Thompson G.G., Goldberg A. (1981). Treatment with haematin in acute hepatic porphyria. QJM.

[74] Pierach C.A., Bossenmaier I., Cardinal R., Weimer M., Watson C.J. (1980). Hematin therapy in porphyric attacks. Klin. Wochenschr..

[75] Pierach C.A. (1982). Hematin therapy for the porphyric attack. Semin. Liver Dis..

[76] Kuo H.C., Lee M.J., Chuang W.L., Huang C.C. (2007). Acute intermittent porphyria with peripheral neuropathy: A follow-up study after hematin treatment. J. Neurol. Sci..

[77] Attarian S., Yu C., Anderson K.E., Friedman E.W. (2017). Effects of hemin and hemodialysis in a patient with acute intermittent porphyria and renal failure. Blood Adv..

[78] Mustajoki P., Tenhunen R., Tokola O., Gothoni G. (1986). Haem arginate in the treatment of acute hepatic porphyrias. BMJ.

[79] Herrick A., McColl K.E., Mclellan A., Moore M.R., Brodie M.J., Goldberg A. (1987). Effect of haem arginate therapy on porphyrin metabolism and mixed function oxygenase activity in acute hepatic porphyria. Lancet.

[80] Tokola O., Mustajoki P., Himberg J.J. (1988). Haem arginate improves hepatic oxidative metabolism in variegate porphyria. Br. J. Clin. Pharmacol..

[81] Volin L., Rasi V., Vahtera E., Tenhunen R. (1988). Heme arginate: Effects on hemostasis. Blood.

[82] Herrick A.L., McColl K.E., Moore M.R., Cook A., Goldberg A. (1989). Controlled trial of haem arginate in acute hepatic porphyria. Lancet.

[83] Kostrzewska E., Gregor A., Tarczyńska-Nosal S. (1991). Heme arginate (Normosang) in the treatment of attacks of acute hepatic porphyrias. Mater. Med. Pol. Pol. J. Med. Pharm..

[84] Mustajoki P., Nordmann Y. (1993). Early administration of heme arginate for acute porphyric attacks. Arch. Intern. Med..

[85] Muthane U.B., Vengamma B., Bharathi K.C., Mamatha P. (1993). Porphyric neuropathy: Prevention of progression using haeme-arginate. J. Intern. Med..

[86] Ma E., Mar V., Varigos G., Nicoll A., Ross G. (2011). Haem arginate as effective maintenance therapy for hereditary coproporphyria. Australas. J. Dermatol..

[87] Lahiji A.P., Anderson K.E., Chan A., Simon A., Desnick R.J., Ramanujam V.M.S. (2020). 5-Aminolevulinate dehydratase porphyria: Update on hepatic 5-aminolevulinic acid synthase induction and long-term response to hemin. Mol. Genet. Metab..

[88] Chapman E., Leal D., Matijasevic E., García E. (2019). Desensitization in patients with hypersensitivity to haem arginate: A case report. World Allergy Organ. J..

[89] Dover S.B., Graham A., Fitzsimons E., Moore M.R., Mccoll K.E.L. (1991). Haem-arginate plus tin-protoporphyrin for acute hepatic porphyria. Lancet.

[90] Isenschmid M., König C., Fässli C., Haenel A., Hänggi W., Schneider H. (1992). Akute intermittierende Porphyrie in der Schwangerschaft: Therapie mit Glukose oder Hämatin? Acute intermittent porphyria in pregnancy: Glucose or hematin therapy?. Schweiz. Med. Wochenschr..

[91] Kuo H., Lin C., Tang Y. (2021). Prophylactic Heme Arginate Infusion for Acute Intermittent Porphyria. Front. Pharmacol..

[92] Yarra P., Faust D., Bennett M., Rudnick S., Bonkovsky H.L. (2019). Benefits of prophylactic heme therapy in severe acute intermittent porphyria. Mol. Genet. Metab. Rep..

[93] Anderson K.E., Collins S. (2006). Open-label study of hemin for acute porphyria: Clinical practice implications. Am. J. Med..

[94] Bonkovsky H.L., Maddukuri V.C., Yazici C., Anderson K.E., Bissell D.M., Bloomer J.R., Phillips J.D., Naik H., Peter I., Baillargeon G. (2014). Acute porphyrias in the USA: Features of 108 subjects from porphyrias consortium. Am. J. Med..

[95] Marsden J.T., Guppy S., Stein P., Cox T.M., Badminton M., Gardiner T., Barth J.H., Stewart M.F., Rees D.C. (2015). Audit of the Use of Regular Haem Arginate Infusions in Patients with Acute Porphyria to Prevent Recurrent Symptoms. JIMD Rep..

[96] Schmitt C., Lenglet H., Yu A., Delaby C., Benecke A., Lefebvre T., Letteron P., Paradis V., Wahlin S., Sandberg S. (2018). Recurrent attacks of acute hepatic porphyria: Major role of the chronic inflammatory response in the liver. J. Intern. Med..

[97] Willandt B., Langendonk J.G., Biermann K., Meersseman W., D’Heygere F., George C., Verslype C., Monbaliu D., Cassiman D. (2016). Liver Fibrosis Associated with Iron Accumulation Due to Long-Term Heme-Arginate Treatment in Acute Intermittent Porphyria: A Case Series. JIMD Rep..

[98] Connolly M.P., Kotsopoulos N., Vermeersch S., Patris J., Cassiman D. (2021). Estimating the broader fiscal consequences of acute hepatic porphyria (AHP) with recurrent attacks in Belgium using a public economic analytic framework. Orphanet J. Rare Dis..

[99] Neeleman R.A., Wagenmakers M., Koole-Lesuis R.H., Mijnhout G.S., Wilson J., Friesema E., Langendonk J.G. (2018). Medical and financial burden of acute intermittent porphyria. J. Inherit. Metab. Dis..

[100] Blaylock B., Epstein J., Stickler P. (2020). Real-world annualized healthcare utilization and expenditures among insured US patients with acute intermittent porphyria (AIP) treated with hemin. J. Med. Econ..

[101] Bonkowsky H.L., Sinclair P.R., Sinclair J.F. (1979). Hepatic heme metabolism and its control. Yale J. Biol. Med..

[102] Waxman A.D., Collins A., Tschudy D.P. (1966). Oscillations of hepatic δ-aminolevulinic acid synthetase produced in, vivo by heme. Biochem. Biophys. Res. Commun..

[103] Elbashir S.M., Harborth J., Lendeckel W., Yalcin A., Weber K., Tuschl T. (2001). Duplexes of 21-nucleotide RNAs mediate RNA interference in cultured mammalian cells. Nature.

[104] Yasuda M., Gan L., Chen B., Kadirvel S., Yu C., Phillips J.D., New M.I., Liebow A., Fitzgerald K., Querbes W. (2021). RNAi-mediated silencing of hepatic Alas1 effectively prevents and treats the induced acute attacks in acute intermittent porphyria mice. Proc. Natl. Acad. Sci. USA.

[105] Vassiliou D., Sardh E., Harper P., Simon A.R., Clausen V.A., Najafian N., Robbie G.J., Agarwal S. (2021). A Drug-Drug Interaction Study Evaluating the Effect of Givosiran, a Small Interfering Ribonucleic Acid, on Cytochrome P450 Activity in the Liver. Clin. Pharmacol. Ther..

[106] Balwani M., Sardh E., Ventura P., Peiró P.A., Rees D.C., Stölzel U., Bissell D.M., Bonkovsky H.L., Windyga J., Anderson K.E. (2020). Phase 3 Trial of RNAi Therapeutic Givosiran for Acute Intermittent Porphyria. New Engl. J. Med..

[107] Ventura P., Bonkovsky H.L., Gouya L., Aguilera-Peiró P., Montgomery Bissell D., Stein P.E., Balwani M., Anderson D., Parker C., Kuter D.J. (2022). Envision Investigators. Efficacy and safety of givosiran for acute hepatic porphyria: 24-month interim analysis of the randomized phase 3 ENVISION study. Liver Int..

[108] Lazareth H., Poli A., Bignon Y., Mirmiran A., Rabant M., Cohen R., Schmitt C., Puy H., Karras A., Gouya L. (2021). Renal Function Decline With Small Interfering RNA Silencing Aminolevulinic Acid Synthase 1 (ALAS1). Kidney Int. Rep..

[109] Petrides P.E., Klein M., Schuhmann E., Torkler H., Molitor B., Loehr C., Obermeier Z., Beykirch M.K. (2021). Severe homocysteinemia in two givosiran-treated porphyria patients: Is free heme deficiency the culprit?. Ann. Hematol..

[110] To-Figueras J., Wijngaard R., García-Villoria J., Aarsand A.K., Aguilera P., Deulofeu R., Brunet M., Gómez-Gómez À., Pozo O.J., Sandberg S. (2021). Dysregulation of homocysteine homeostasis in acute intermittent porphyria patients receiving heme arginate or givosiran. J. Inherit. Metab. Dis..

[111] Ricci A., Marcacci M., Cuoghi C., Pietrangelo A., Ventura P. (2021). Hyperhomocysteinemia in patients with acute porphyrias: A possible effect of ALAS1 modulation by siRNAm therapy and its control by vitamin supplementation. Eur. J. Intern. Med..

[112] Fontanellas A., Ávila M.A., Arranz E., Enríquez de Salamanca R., Morales-Conejo M. (2021). Acute intermittent porphyria, givosiran, and homocysteine. J. Inherit. Metab. Dis..

[113] Vassiliou D., Sardh E. (2021). Homocysteine elevation in givosiran treatment: Suggested ALAS1 siRNA effect on cystathionine beta-synthase. J. Intern. Med..

[114] Ventura P., Sardh E., Longo N., Balwani M., Plutsky J., Gouya L., Philipps J., Rhyee S., Fanelli M.-J., Sweetser M.T. (2022). Hyperhomocysteinemia in acute hepatic porphyria (AHP) and implications for treatment with givosiran.

[115] Poli A., Schmitt C., Moulouel B., Mirmiran A., Talbi N., Rivière S., Cerutti D., Bouchoule I., Faivre A., Grobost V. (2022). Givosiran in acute intermittent porphyria: A personalized medicine approach. Mol. Genet. Metab..

[116] Hausdorf G., Roggenbuck D., Feist E., Büttner T., Jungblut P.R., Conrad K., Berg C., Klein R. (2009). Autoantibodies to asialoglycoprotein receptor (ASGPR) measured by a novel ELISA—Revival of a disease-activity marker in autoimmune hepatitis. Clin. Chim. Acta.

[117] Massachi S., Epstein J., Hurd J., Bonkovsky H.L. (2020). Cost savings with hemin versus givosiran for the treatment of patients with acute intermittent porphyria (AIP). J. Med. Econ..

[118] Sardh E., Rejkjaer L., Andersson D.E., Harper P. (2007). Safety, pharmacokinetics and pharmocodynamics of recombinant human porphobilinogen deaminase in healthy subjects and asymptomatic carriers of the acute intermittent porphyria gene who have increased porphyrin precursor excretion. Clin. Pharmacokinet..

[119] Córdoba K.M., Serrano-Mendioroz I., Jericó D., Merino M., Jiang L., Sampedro A., Alegre M., Corrales F., Garrido M.J., Martini P. (2022). Recombinant porphobilinogen deaminase targeted to the liver corrects enzymopenia in a mouse model of acute intermittent porphyria. Sci. Transl. Med..

[120] Jiang L., Berraondo P., Jericó D., Guey L.T., Sampedro A., Frassetto A., Benenato K.E., Burke K., Santamaría E., Alegre M. (2018). Systemic messenger RNA as an etiological treatment for acute intermittent porphyria. Nat. Med..

[121] Bustad H.J., Kallio J.P., Vorland M., Fiorentino V., Sandberg S., Schmitt C., Aarsand A.K., Martinez A. (2021). Acute Intermittent Porphyria: An Overview of Therapy Developments and Future Perspectives Focusing on Stabilisation of HMBS and Proteostasis Regulators. Int. J. Mol. Sci..

